# Effect of Palmitic Acid on Exosome-Mediated Secretion and Invasive Motility in Prostate Cancer Cells

**DOI:** 10.3390/molecules25122722

**Published:** 2020-06-12

**Authors:** Ivan V. Maly, Wilma A. Hofmann

**Affiliations:** Department of Physiology and Biophysics, Jacobs School of Medicine and Biomedical Sciences, State University of New York at Buffalo, Buffalo, NY 14203, USA; ivanmaly@buffalo.edu

**Keywords:** palmitic acid, fatty acids, prostate cancer, migration, invasion, exosomes, caveolin-1, secretion

## Abstract

High fat consumption can enhance metastasis and decrease survival in prostate cancer, but the picture remains incomplete on the epidemiological and cell-biological level, impeding progress toward individualized recommendations in the clinic. Recent work has highlighted the role of exosomes secreted by prostate cancer cells in the progression of the disease, particularly in metastatic invasion, and also the utility of targeting these extracellular vesicles for diagnostics, as carriers of disease progression markers. Here, we investigated the question of a potential impact of the chief nutritional saturated fatty acid on the exosome secretion. Palmitic acid decreased the secretion of exosomes in human prostate cancer cells in vitro in a concentration-dependent manner. At the same time, the content of some prospective metastatic markers in the secreted exosomal fraction was also reduced, as was the ability of the cells to invade across extracellular matrix barriers. While by themselves our in vitro results imply that on the cell level, palmitic acid may be beneficial vis-à-vis the course of the disease, they also suggest that, by virtue of the decreased biomarker secretion, palmitic acid has the potential to cause unjustified deprioritization of treatment in obese and lipidemic men.

## 1. Introduction

High-fat diet, and specifically diet rich in palmitic acid [1,2], has been epidemiologically associated with a risk of prostate cancer [3,4,5]. At the same time, mortality in prostate cancer is related to metastasis [6,7]. These facts draw attention to the potential for suppressing the disease’s progression to lethal forms by dietary intervention post-diagnosis [8,9]. Indeed, mouse models of prostate cancer have shown a correlation of poor prognosis and specifically metastasis with a high-fat diet [10,11]. An additional important aspect of changes associated with a high-fat diet in the prostate and other tissues is the generation of reactive oxygen species via the upregulated nicotinamide adenine dinucleotide phosphate (NADPH) oxidase system and activation of nuclear factor κB (NF-κB) signaling that leads to chronic inflammation and cancerogenesis [12,13,14,15]. As palmitic acid is the major or chief component of the Western-style high-fat diet, studies of its impact on the metastatic potential of prostate cancer cells may shed light on the plausibility of such intervention regimens or an antilipidemic approach.

The nutritional concerns dovetail with the increasingly evident role of lipids in the biogenesis and activity of prostate cell-derived exosomes. Studies have demonstrated that exosome-mediated cell–cell communication and functions, such as cell invasivity, play an important part in the progression of prostate cancer, while exosome-associated biomarkers gain acceptance in diagnostics [16,17,18,19,20,21]. Our own recent work, in particular, showed that the secretion of matrix metalloprotease-containing exosomes with the participation of a myosin isoform specific to advanced prostate cancer is accompanied by enhanced invasivity of the cells across extracellular matrix barriers [22,23,24].

Other recent experiments on prostate cancer cells revealed that the secretion of exosomes by prostate cancer cells and their bioactive content, such as vascular endothelial growth factor, depends critically on the availability of plasma-complexed lipids [18], and multiple studies have addressed the reflection of specific available lipids on the exosomes’ biogenesis pathways, which lead to differentiated bioactive content, for example, the exosome-bound microRNA (miRNA) [25,26]. At the same time, given the diversity of the biomedical and biochemical contexts where exosomes play a role, it may be accepted that the impact of major individual lipids on exosomes’ secretion and that of non-lipid exosome-associated molecules remains an under-investigated area.

In the present work, we sought to add to the reviewed literature by investigating the impact of supplementation with the dominant free fatty acid, palmitic acid, on exosome secretion and exosome-associated functions of prostate cancer cells, such as secretion of progression biomarkers and invasive motility. The observed inhibition of the secretion of exosomes, secretion of exosome-associated biomarkers, and invasive motility by palmitic acid suggests a potentially complex impact of this major nutrient on the progression and prognostic monitoring of prostate cancer, whereby the individually beneficial cell-level response may become offset by an increased difficulty in second-line treatment prioritization.

## 2. Results

**Figure 1:**
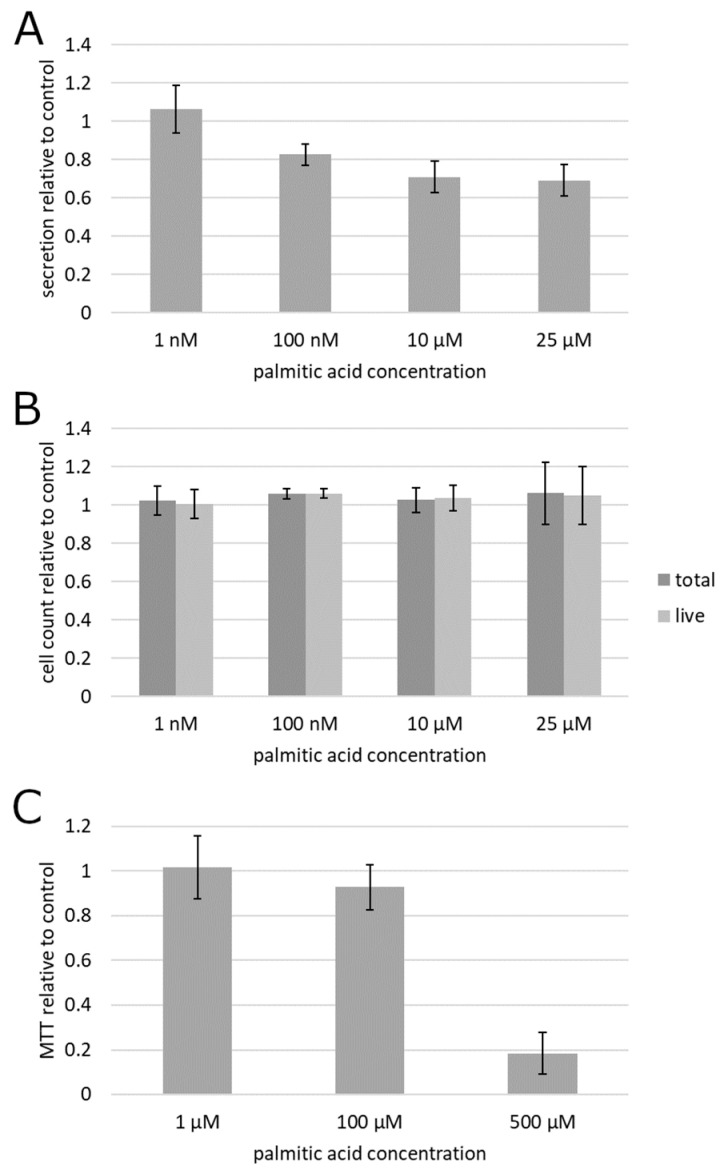
Effect of palmitic acid on exosome secretion and cell survival and proliferation in prostatic carcinoma PC3 cells. (**A**) Fluorescent measurement of the exosomal fraction of the culture supernatant. (**B**) Cell count. (**C**) Metabolic colorimetric MTT assay. Error bars, 95% confidence interval. Each plotted value is the mean of at least 6 biological replicates (cell cultures) from at least 2 different culture passage numbers.

To begin testing the impact of albumin-complexed palmitic acid on cultured prostate cancer cells, the culture supernatant of prostatic carcinoma (PC3) cells treated by the albumin preparations with and without palmitic acid was subjected to a centrifugation-based protocol for separation of exosomes. Fluorescent labeling of lipid membranes in the exosomal fraction revealed that exosome secretion was suppressed by palmitic acid beginning with as low as 100 nM (Figure 1A). This result was statistically significant on the significance level 0.05 as determined by the confidence interval method. To establish whether the reduced exosome secretion could be explained by a cytotoxic or anti-proliferative action of palmitic acid, rather than through a mechanism more specific to exosome secretion, the cells were treated the same way and counted. The data showed that the exosome secretion changes were not accompanied by any measurable effect on cell numbers or viability (Figure 1B). Although the results obtained at 100 nM, in contrast to the other concentrations, displayed a formally statistically significant difference from the albumin control as determined at the significance level 0.05 in the confidence interval method, the mean increase of 6% in both total and live cell counts could be deemed insubstantial.

To further analyze the effect of palmitic acid on metabolic activity in the cell culture, the 3-dimethylthiazolyl-2,5-diphenyltetrazolium (MTT) colorimetric assay for mitochondrial activity was conducted (Figure 1C). It showed a pronounced and statistically significant (*p* < 0.05 by the confidence interval method) suppression, relative to the albumin control, of cell culture metabolism at 500 µM (far outside the range tested in other experiments reported here), and no statistically significant (α = 0.05) alteration at 1 and 100 µM, indicating that exosome secretion changes are not accompanied by changes in metabolic activity (Figure 1C).

**Figure 2:**
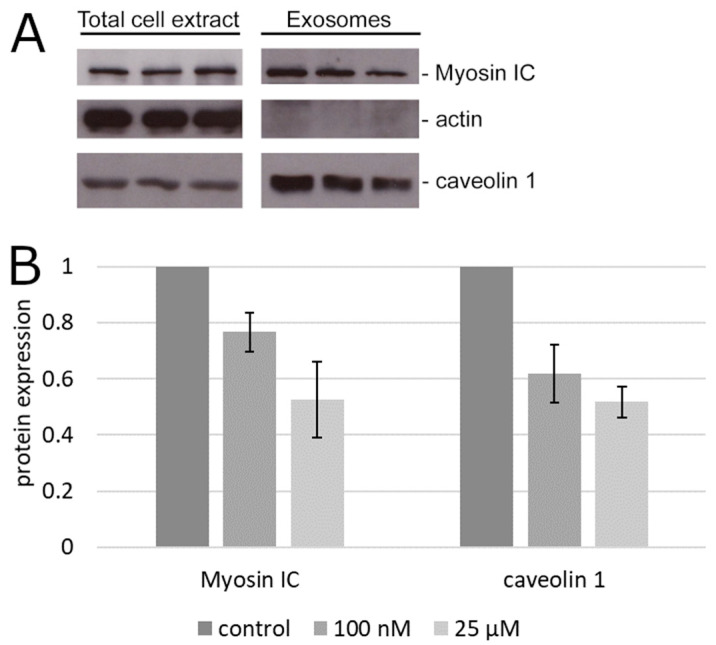
Content of advanced prostate cancer markers in the exosomal fraction. (**A**) Representative immunoblot of total cell extract and secreted exosomes. The protein content in the total extract of the adherent cells is shown for comparison with the exosomal fraction of the supernatant from the same cultures. (**B**) Densitometry of the results in (**A**). Error bars, standard error of the mean.

To determine if the reduction of bulk exosome secretion was associated with any impact on the secretion of specific exosome-associated markers of prostate cancer, we tested the exosomal fraction of the cell culture supernatant of PC3 cells for the content of two proteins that are both involved in exosome secretion and implicated in prostate cancer progression but otherwise widely different, caveolin 1 [19,20,21] and myosin IC [22,23,27]. The secreted exosomal caveolin 1 was reduced significantly (*p* < 0.05 by the confidence interval method) relative to the albumin control by palmitic acid already at 100 nM (Figure 2). Similar results were obtained with myosin IC (Figure 2).

**Figure 3:**
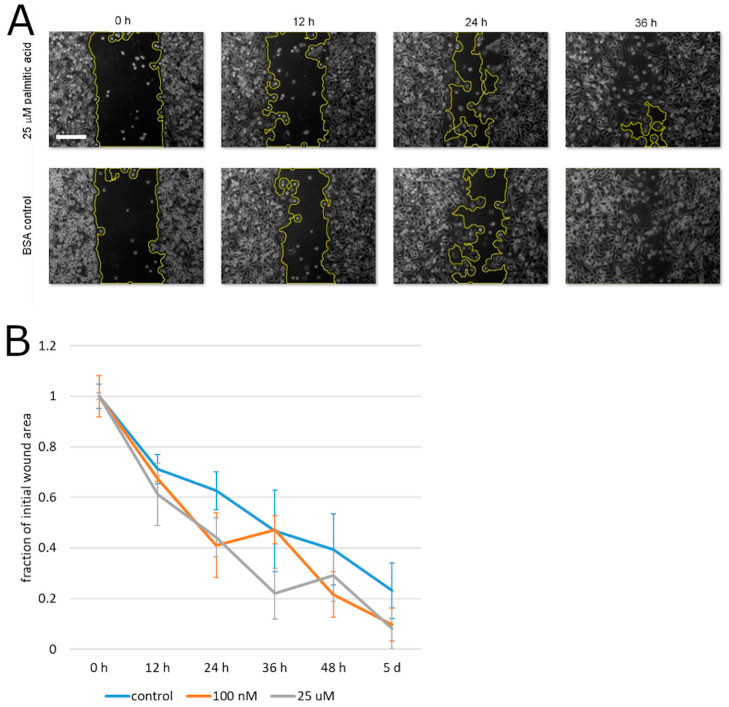
PC3 cell migration in a monolayer wound healing assay. (**A**) Representative microscopic images showing the wound closure by PC3 cells in the presence of 25 µM palmitic acid and in the albumin carrier control experiment. Wound area outline in yellow is overlaid by the software used for the automated area measurement. Scale bar: 200 µm. (**B**) Analysis of the experimental wound closure. Error bars, standard error on the mean. Each plotted value is the mean of 4 independent experiments.

**Figure 4:**
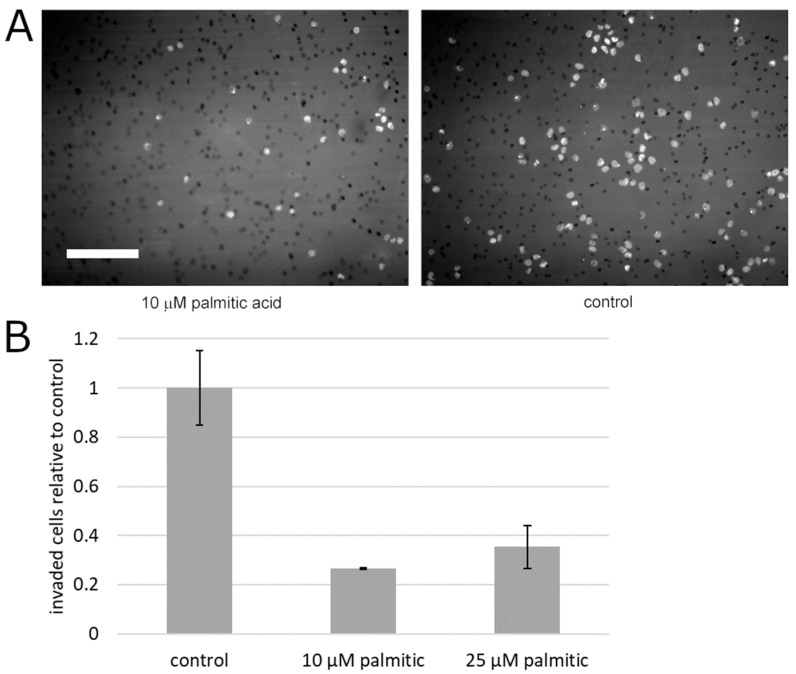
Palmitic acid significantly reduces extracellular matrix invasion by PC3 cells. (**A**) Representative microscopic images of 4′-6-diamidino-2-phenylindole (DAPI)-stained PC3 cells on the bottom surface of the Matrigel-sealed transwell in the presence of 10 µM palmitic acid or the albumin carrier control. Bright spots are the cell nuclei and small dark circles are the pores in the transwell membrane. Scale bar: 200 µm. (**B**) Analysis of the number of invasive cells. Error bars, standard error on the mean. Each plotted value is the mean of 3 independent experiments. Nine microscopic fields covering the area of the transwell were acquired and quantified in each experiment, per treatment condition.

To begin testing the effect of palmitic acid on the motility of prostate cancer cells, we measured the capacity of PC3 cells for migration that is unimpeded by the extracellular matrix. To this aim, the cells’ motility was analyzed in the monolayer wound assay. During the closure of the experimental wound over the course of up to 5 days, palmitic acid did not cause any statistically significant deviation between the albumin control and the same concentrations of palmitic acid that caused the above-documented effects (0.1–25 µM, Figure 3). Specifically, at all time points, the differences between the treatment groups and the control were not significant on the significance level 0.05 by the confidence interval method. This negative result notwithstanding, we assessed the effect of palmitic acid on the ability of prostate cancer cells to migrate across an extracellular matrix barrier. PC3 cells were exposed to 10 and 25 µM palmitic acid and assayed using the modified Boyden chamber method. The counts of the cells on the other side of the Matrigel layer 24 h after plating were found to be reduced compared to cells treated with the corresponding concentration of the albumin carrier alone (Figure 4). The reduction was approximately four-fold and significant on the significance level *α* = 0.05.

## 3. Discussion

The new results demonstrate that when delivered in the complex with serum albumin in vitro, palmitic acid suppresses the secretion of exosomes and exosome-associated molecules characteristic of prostate cancer. This effect is accompanied by a decreased ability of prostate cancer cells to invade across extracellular matrix barriers. On the whole, the results suggest that while a reduction of palmitic acid intake may not be beneficial to the course of the disease, the movement toward the adoption of exosome-associated prognostic markers may necessitate carefully weighed prioritization of second-line treatment in lipidemic men.

The results add to the recent literature on the effect lipids have on the secretion and composition of prostate cancer-associated and other exosomes. It has been known that delipidization of serum in the culture medium, in addition to lipid metabolism inhibition, suppresses exosome production and loading with vascular endothelial growth factor by prostate cancer cells [18]. Direct extrapolation from the cited work might suggest that adding a major lipid (free fatty acid) to the medium should have a positive effect on the exosome secretion. However, in the new experiments on PC3 cells, we observed the opposite effect that was dose dependent already at comparatively low concentrations, at or below 25 µM—the physiological free albumin-complexed palmitic acid concentration reaching at least 100 µM in humans [28]. In combination, the existing data suggest that the rate of exosome secretion in advanced human prostate cells is sensitive to palmitic acid. To delineate the effect on exosome-mediated secretion from any general suppression of cell viability or proliferation, we ascertained that neither the cell count nor the metabolic rate was affected by the palmitic acid treatments that caused the secretion reduction. At the same time, we note that a higher concentration can promote proliferation of cultured prostate cancer cells, as demonstrated by the recent work [29], where 100 µM albumin-coupled palmitic acid was tested and found to increase the cells’ proliferation by approximately 10%.

Recent work has painted a considerably detailed portrait of how various lipids (e.g., cholesterol, ceramides, or phosphatidylinositol trisphosphate PIP3) affect the lipid makeup of the secreted exosomes [25]. Additionally, high fat consumption in mice leads to palmitic acid accumulation and relative polyunsaturated fatty acid depletion in tissues, accompanied by upregulation of resolvins, which can confound the inflammatory picture on the background of the oxidative stress [30]. Non-lipid bioactive exosome components are also impacted, as in the case of the ceramide-dependent production of miRNA-containing exosomes [26]. Our results add to this picture by highlighting how, in prostate cancer cells, palmitic acid determines the exosome-associated content of the metastatic progression marker and exosomal secretion molecule caveolin 1 [19,20,21,22] and prostate cancer-associated exosomal motor protein myosin IC [22,23,27]. The new results suggest that not only can the cell–cell communications function of exosomes, documented in the progression of prostate cancer [17,21,31,32], be regulated by palmitic acid but also the use of the prostate-derived exosome-associated markers of tumor progression can become quantitatively skewed depending on the individual’s lipid intake and metabolism. The latter speculation is, obviously, a wide extrapolation from the results obtained in a reduced system in vitro. Nevertheless, this appears to be a hypothesis worth testing in an epidemiologic and nutritional cohort study.

In light of the recently established association of exosome secretion with the invasive ability of prostate cancer cells [17,18,22], we complemented the tests of the secretion by addressing the effect of palmitic acid in cell motility assays, primarily employing the modified Boyden chamber method to test for cells’ invasive ability. We also conducted monolayer wound experiments to aid in the differentiation of the Boyden chamber results as having to do functionally with motility per se or narrowly with cell–matrix interactions. No pronounced influence was seen in the monolayer wound assay, thus establishing a background for the interpretation of subsequent Matrigel-modified Boyden chamber experiments. Although we classified our monolayer wound result as negative, it is quantitatively compatible with the recent demonstration of a stimulating effect of palmitic acid in the same assay [29]. The prior communication reported an enhancement of migration that resulted after 48 h of exposure to 100 µM palmitic acid, in the wound area closure to approximately 5% of the initial value, compared with approximately 12.5% for untreated cells. By comparison, here we traced the time course of the monolayer wound closure at 12-h intervals and up to 5 days at palmitic acid concentrations up to 25 µM (Figure 3), and observed no effect, with the closure followed to approximately 10–20% of the initial wound area, whose magnitude could furnish an explanation for the marked invasion suppression. While the exact regimen of fatty acid exposure in vitro will be fine-tuned in future experiments, the new results add new data points to the picture of physiological responses on the cellular level arising from the cited work.

**Figure 5:**
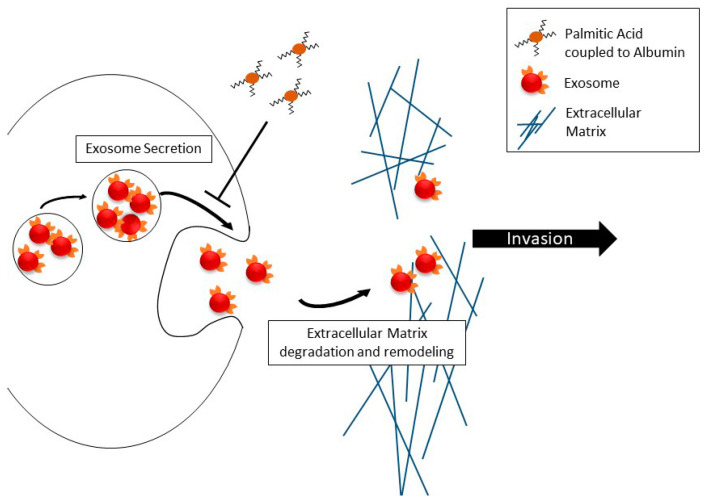
Schematic representation of the involvement of exosome secretion in invasive motility and its regulation by albumin-complexed palmitic acid.

Cell motility as such is only one functional component of migration across extracellular matrix barriers, with the most notable other component being digestion and remodeling of the extracellular matrix, which enables motile cells’ progress through the meshwork of basement membrane proteins, which is impenetrable to differentiated epithelial cells under normal conditions. The digestion and remodeling in metastatic prostate cancer cells is associated with secreted matrix metalloproteases [16,17,22]. We recently found that in prostate cancer cells, free (unimpeded) migration and invasion across the reconstituted extracellular matrix are controlled differently by isoforms of myosin IC [22]. While myosin IC controls unimpeded cell migration, the advanced prostate cancer-specific isoform A [23,24] of this motor protein controls invasion by promoting the secretion of exosomes containing matrix metalloproteases. Thus, building on the cited recent work that analyzed the effect of palmitic acid on unimpeded migration [29], our new findings in the in vitro extracellular matrix model (Figure 4) show that dissemination of metastatic prostate cancer cells from the primary site exhibits another layer of palmitic acid-regulated complexity over cell locomotion. Our new experiments show that the net effect may be a marked reduction of the cells’ ability to invade dense tissues. In light of the other results concerning matrix metalloproteases and exosomes, including our recent work on prostate cancer cell invasion [22], and the reduction of secretion reported here, we attribute the reduced ability to invade to the reduction in exosome secretion. The schematic in Figure 5 encapsulates this interpretation. Additional experiments will be needed to directly test it.

Summarizing, the data in this report show that the major fatty acid component of the high-fat Western diet, palmitic acid, suppresses the secretion of exosomes and exosome-associated molecules in prostate cancer cells, suggesting a possible impact of lipidemia on the prioritization of second-line treatment in obese men. The cell-level effect of palmitic acid appears to be a reduction of the capacity of the cells for metastatic dissemination, pointing to higher-level organismal functions as being more likely explanations for the enhanced metastasis seen in the prior mouse models of high fat consumption. In human patients, additionally, the cell-level inhibition of the key step to disease progression may be offset by an increased difficulty in assessing the progression using secreted serum markers. Although this supposition is speculative, based on narrow in vitro data, its implications suggest that it may merit further investigation.

## 4. Materials and Methods

### 4.1. Cell Culture and Cell Counting

PC3 cells [33] were purchased from ATCC (American Type Culture Collection, Manassas, VA, USA, catalog number CRL-1435, purchase date 01/2014). The cells were acquired from the repository directly. The cultures were maintained in RPMI 1640 medium without phenol red (Invitrogen, Carlsbad, CA, USA) supplemented with 10% fetal bovine serum and 1% penicillin/streptomycin, at 37 °C with 5% CO_2_. Cells were counted after lifting with trypsin ethylenediaminetetraacetic acid (EDTA) solution suitable for cell culture (Sigma-Aldrich, St. Louis, MO, USA) and staining with 0.4% trypan blue (Thermo Fisher, Waltham, MA, USA), using an automatic cell counter (TC20, Bio-Rad, Hercules, CA, USA).

### 4.2. Treatment with Palmitic Acid

Fatty-acid-free bovine serum albumin (BSA), catalog number A7030, and palmitic acid, suitable for cell culture, ≥99% pure, were purchased from Sigma-Aldrich (St. Louis, MO, USA). Palmitic acid was complexed with fatty-acid-free BSA at a 5:1 molar ratio. Palmitic acid was dissolved and saponified in 0.1 M NaOH at 70 °C. Fatty-acid-free BSA was reconstituted in 150 mM NaCl to obtain a 2 mM BSA solution and kept warm at 37 °C. Palmitic acid was then added to obtain a 10 mM fatty acid, 2 mM BSA stock solution. The solution was incubated for 2 h at 37 °C with vigorous shaking to encourage coupling of the fatty acid to BSA. The stock solution was kept at −20 °C. The control BSA solution was prepared identically except that palmitic acid was absent from the preparation.

For treatment, cells were seeded and incubated for 24 h in culture medium. Before treatment, the culture medium was removed, and the cells were equilibrated for 2 h in serum-free RPMI 1640 medium. The equilibration medium was then replaced by serum-free RPMI 1640 medium enriched with the BSA-coupled palmitic acid at the indicated concentration (100 nM through 500 µM) for 48 h. Control cells received medium with fatty-acid-free BSA at equal concentrations to the palmitic acid-coupled BSA in the experimental wells.

### 4.3. Exosome Separation and Quantification

Isolation and analysis of exosomes were performed as described previously [22]. In brief, 0.8 × 10^6^ cells were plated in 10 cm tissue culture dishes in complete culture medium. Then, 24 h after plating, cells were treated as described above. After 48 h, the supernatant was collected, cleared of cell debris, and subjected to serial ultracentrifugation for exosome isolation. Specifically, 10 mL of the supernatant were centrifuged at 500× *g* at room temperature for 10 min. The supernatant from the last step was transferred to a new tube and further centrifuged at 20,000× *g* for 30 min at 4 °C. The supernatant from the last step was transferred to a new tube and further centrifuged at 100,000× *g* for 90 min at 4 °C. The supernatant from the last step was discarded and the pellet resuspended in 500 µL of Vybrant DiI (Thermo Fisher, Waltham, MA, USA) solution that was prepared in PBS according to the manufacturer’s instructions. Following incubation in the dark for 20 min at 37 °C, 10 mL of PBS were added, and the last centrifugation step was repeated. The pelleted stained exosomes were resuspended in 10 mL of PBS for the measurements. The adherent cells from the same cultures were counted as described above and subjected to routine whole-cell extraction as described previously [22] in order to confirm that the secreted exosomes arose from a comparable amount of cell culture.

### 4.4. Immunoblotting

The detection of proteins in the exosomal fraction was performed as described previously [22]. Briefly, the proteins were separated by SDS-PAGE and transferred onto nitrocellulose membrane. After the transfer, the membranes were cut according to the size of the protein and probed with specific antibodies: Anti-caveolin 1 (Cell Signaling Technologies, Danvers, MA, USA), anti-myosin IC (Santa Cruz Biotechnology, Dallas, TX, USA), and anti-β-actin (Sigma-Aldrich, St. Louis, MO, USA). The immunoreactive bands were detected by enhanced chemiluminescence. Densitometry for expression quantification was performed in Image J (National Institutes of Health, Bethesda, MD, USA). The adherent cells from the same cultures were counted as described above and subjected to routine whole-cell extraction as described previously [22] in order to confirm that the secreted exosomes arose from a comparable amount of cell culture.

### 4.5. Invasion Assay

The Matrigel-modified Boyden chamber transwell assay [34,35] was employed as before [22] to assess the capacity of cells for invasion across extracellular matrix barriers. The cells were seeded on top of a Matrigel invasion chamber (Corning, Bedford, MA, USA) in equal numbers (2.5 × 10^4^ per chamber) following the same treatment as described above, and in the same treatment solutions. Following a 24-h incubation (37 °C, 5% CO_2_), the Matrigel and cells remaining on the top surface of the well bottom were removed by scraping, and the cells on the bottom surface were fixed in 3% paraformaldehyde in PBS. The porous membrane forming the well bottom was separated from the well and mounted in the 4′,6-diamidino-2-phenylindole (DAPI)-containing medium (Prolong, Cell Signaling Technologies, Danvers, MA, USA) for fluorescent visualization of the nuclei. Nonoverlapping fields covering the area of the membrane were acquired with a charge-coupled device (CCD) camera (RT Slider, SPOT Imaging, Sterling Heights, MI, USA) above an upright microscope (DMRE, Leica, Wetzlar, Germany). The nuclei in each field were detected automatically and counted in Image J using the Analyze Particles utility, and the number was accepted as a cell count. Identical algorithm parameters were used on all images.

### 4.6. Migration Assay

The in vitro cell culture monolayer wound (scratch) healing assay [36,37] was employed as before [22] to assess the capacity of cells for locomotion that is unimpeded by extracellular matrix. Following the same palmitic acid and control treatment as described above, PC3 cells were seeded in 6-well plates (Costar treated for tissue culture, Corning, Bedford, MA, USA) at equal densities in the treatment solution (not containing any serum). Then, 24 h later, the monolayer of cells was scratched with a 200-µL pipette tip, producing a “wound” that was clear of cells and had a uniform width narrower than the microscope’s camera’s field of view. The wounded monolayers were washed with PBS to remove any cell debris and the wells were re-filled with fresh identical treatment solution. The closing wound area was photographed at indicated time points past the time of the scratch. Each time, the culture was washed with PBS before imaging, and the wells refilled with fresh identical treatment solution. Nonoverlapping fields covering the length of the wound were acquired on an inverted microscope (DMIL, Leica, Wetzlar, Germany) using a CCD camera (RT Slider, SPOT Imaging, Sterling Heights, MI, USA). The wound area (area free of cells) in each field was detected automatically and measured in the Image J software using the MRI Wound Healing Tool utility. Identical algorithm parameters were used on all images, and the measurement was conducted using the same method at all time points.

### 4.7. 3-Dimethylthiazolyl-2,5-diphenyltetrazolium (MTT) Assay

Cell proliferation and survival was assessed by the MTT colorimetric method [38] according to the manufacturer’s instructions and as described previously [39]. MTT bromide suitable for cell culture was purchased from Sigma-Aldrich (St. Louis, MO, USA) and reconstituted in PBS to obtain a stock solution of 5 mg/mL. After cell treatment (described above), the culture medium was removed from the wells and replaced with an equal volume of MTT treatment solution. The MTT treatment solution was prepared by adding 10% (by volume) of the MTT stock solution to RPMI 1640 without phenol red. The cells were incubated with the MTT treatment solution for 1 h at 37 °C under 5% CO_2_. The solution was then removed, and 100 μL of dimethyl sulfoxide (DMSO) were added to each well to solubilize the MTT formazan crystals. The plate was incubated for 20 min on an orbital shaker with agitation, and the absorbance was measured at 540 nm using a BioTek Synergy microplate reader (BioTek Instruments, Inc., Winooski, VA, USA). The cell viability was calculated as 100% × (absorbance of treated wells–absorbance of blank control wells)/(absorbance of albumin control wells–absorbance of blank control wells). The blank control wells were treated identically to the other wells, except that no cells were seeded, and the medium was free of BSA and fatty acids. The MTT experiments were performed in triplicate (on three cell culture wells) and additionally repeated on different cell passages.

## References

[1] Kurahashi N., Inoue M., Iwasaki M., Sasazuki S., Tsugane A.S. (2008). Dairy product, saturated fatty acid, and calcium intake and prostate cancer in a prospective cohort of Japanese men. Cancer Epidemiol. Biomark. Prev..

[2] Bassett J.K., Severi G., Hodge A.M., MacInnis R.J., Gibson R.A., Hopper J.L., English D.R., Giles G.G. (2013). Plasma phospholipid fatty acids, dietary fatty acids and prostate cancer risk. Int. J. Cancer.

[3] Di Sebastiano K.M., Mourtzakis M. (2014). The role of dietary fat throughout the prostate cancer trajectory. Nutrients.

[4] Maly I.V., Hofmann W.A. (2018). Fatty acids and calcium regulation in prostate cancer. Nutrients.

[5] Schwingshackl L., Schwedhelm C., Galbete C., Hoffmann G. (2017). Adherence to mediterranean diet and risk of cancer: An updated systematic review and meta-analysis. Nutrients.

[6] Swanson G.P., Basler J.W. (2010). Prognostic factors for failure after prostatectomy. J. Cancer.

[7] Swanson G.P., Chen W., Trevathan S., Hermans M. (2020). Long-term follow-up after prostatectomy for prostate cancer and the need for active monitoring. Prostate Cancer.

[8] Lin P.H., Aronson W., Freedland S.J. (2015). Nutrition, dietary interventions and prostate cancer: The latest evidence. BMC Med..

[9] Lin P.H., Aronson W., Freedland S.J. (2019). An update of research evidence on nutrition and prostate cancer. Urol. Oncol..

[10] Cho H.J., Kwon G.T., Park H., Song H., Lee K.W., Kim J.I., Park J.H. (2015). A high-fat diet containing lard accelerates prostate cancer progression and reduces survival rate in mice: Possible contribution of adipose tissue-derived cytokines. Nutrients.

[11] Chang S.N., Han J., Abdelkader T.S., Kim T.H., Lee J.M., Song J., Kim K.S., Park J.H., Park J.H. (2014). High animal fat intake enhances prostate cancer progression and reduces glutathione peroxidase 3 expression in early stages of TRAMP mice. Prostate.

[12] Shankar E., Bhaskaran N., MacLennan G.T., Liu G., Daneshgari F., Gupta S. (2015). Inflammatory signaling involved in high-fat diet induced prostate diseases. J. Urol. Res..

[13] Li Y., Shi B., Dong F., Zhu X., Liu B., Liu Y. (2019). Effects of inflammatory responses, apoptosis, and STAT3/NF-kappaB- and Nrf2-mediated oxidative stress on benign prostatic hyperplasia induced by a high-fat diet. Aging (Albany NY).

[14] Soto-Alarcon S.A., Ortiz M., Orellana P., Echeverria F., Bustamante A., Espinosa A., Illesca P., Gonzalez-Manan D., Valenzuela R., Videla L.A. (2019). Docosahexaenoic acid and hydroxytyrosol co-administration fully prevents liver steatosis and related parameters in mice subjected to high-fat diet: A molecular approach. Biofactors.

[15] Illesca P., Valenzuela R., Espinosa A., Echeverria F., Soto-Alarcon S., Ortiz M., Videla L.A. (2019). Hydroxytyrosol supplementation ameliorates the metabolic disturbances in white adipose tissue from mice fed a high-fat diet through recovery of transcription factors Nrf2, SREBP-1c, PPAR-gamma and NF-kappaB. Biomed. Pharmacother..

[16] Deep G., Jain A., Kumar A., Agarwal C., Kim S., Leevy W.M., Agarwal R. (2020). Exosomes secreted by prostate cancer cells under hypoxia promote matrix metalloproteinases activity at pre-metastatic niches. Mol. Carcinog..

[17] Ramteke A., Ting H., Agarwal C., Mateen S., Somasagara R., Hussain A., Graner M., Frederick B., Agarwal R., Deep G. (2015). Exosomes secreted under hypoxia enhance invasiveness and stemness of prostate cancer cells by targeting adherens junction molecules. Mol. Carcinog..

[18] Schlaepfer I.R., Nambiar D.K., Ramteke A., Kumar R., Dhar D., Agarwal C., Bergman B., Graner M., Maroni P., Singh R.P. (2015). Hypoxia induces triglycerides accumulation in prostate cancer cells and extracellular vesicles supporting growth and invasiveness following reoxygenation. Oncotarget.

[19] Llorente A., de Marco M.C., Alonso M.A. (2004). Caveolin-1 and MAL are located on prostasomes secreted by the prostate cancer PC-3 cell line. J. Cell Sci..

[20] Mathieu R., Klatte T., Lucca I., Mbeutcha A., Seitz C., Karakiewicz P.I., Fajkovic H., Sun M., Lotan Y., Scherr D.S. (2016). Prognostic value of Caveolin-1 in patients treated with radical prostatectomy: A multicentric validation study. BJU Int..

[21] Syn N., Wang L., Sethi G., Thiery J.P., Goh B.C. (2016). Exosome-mediated metastasis: From epithelial-mesenchymal transition to escape from immunosurveillance. Trends Pharmacol. Sci..

[22] Maly I.V., Domaradzki T.M., Gosy V.A., Hofmann W.A. (2017). Myosin isoform expressed in metastatic prostate cancer stimulates cell invasion. Sci. Rep..

[23] Ihnatovych I., Sielski N.L., Hofmann W.A. (2014). Selective expression of myosin IC Isoform A in mouse and human cell lines and mouse prostate cancer tissues. PLoS ONE.

[24] Ihnatovych I., Migocka-Patrzalek M., Dukh M., Hofmann W.A. (2012). Identification and characterization of a novel myosin Ic isoform that localizes to the nucleus. Cytoskeleton (Hoboken).

[25] Record M., Silvente-Poirot S., Poirot M., Wakelam M.J.O. (2018). Extracellular vesicles: Lipids as key components of their biogenesis and functions. J. Lipid Res..

[26] Kosaka N., Iguchi H., Yoshioka Y., Takeshita F., Matsuki Y., Ochiya T. (2010). Secretory mechanisms and intercellular transfer of microRNAs in living cells. J. Biol. Chem..

[27] Saidova A.A., Potashnikova D.M., Tvorogova A.V., Maly I.V., Hofmann W.A., Vorobjev I.A. (2018). Specific and reliable detection of Myosin 1C isoform A by RTqPCR in prostate cancer cells. PeerJ.

[28] Yli-Jama P., Meyer H.E., Ringstad J., Pedersen J.I. (2002). Serum free fatty acid pattern and risk of myocardial infarction: A case-control study. J. Intern. Med..

[29] Landim B.C., de Jesus M.M., Bosque B.P., Zanon R.G., da Silva C.V., Goes R.M., Ribeiro D.L. (2018). Stimulating effect of palmitate and insulin on cell migration and proliferation in PNT1A and PC3 prostate cells: Counteracting role of metformin. Prostate.

[30] Echeverria F., Valenzuela R., Espinosa A., Bustamante A., Alvarez D., Gonzalez-Manan D., Ortiz M., Soto-Alarcon S.A., Videla L.A. (2019). Reduction of high-fat diet-induced liver proinflammatory state by eicosapentaenoic acid plus hydroxytyrosol supplementation: Involvement of resolvins RvE1/2 and RvD1/2. J. Nutr. Biochem..

[31] Bijnsdorp I.V., Geldof A.A., Lavaei M., Piersma S.R., van Moorselaar R.J., Jimenez C.R. (2013). Exosomal ITGA3 interferes with non-cancerous prostate cell functions and is increased in urine exosomes of metastatic prostate cancer patients. J. Extracell. Vesicles.

[32] Corcoran C., Rani S., O’Brien K., O’Neill A., Prencipe M., Sheikh R., Webb G., McDermott R., Watson W., Crown J. (2012). Docetaxel-resistance in prostate cancer: Evaluating associated phenotypic changes and potential for resistance transfer via exosomes. PLoS ONE.

[33] Kaighn M.E., Narayan K.S., Ohnuki Y., Lechner J.F., Jones L.W. (1979). Establishment and characterization of a human prostatic carcinoma cell line (PC-3). Invest. Urol..

[34] Marshall J. (2011). Transwell((R)) invasion assays. Methods Mol. Biol..

[35] Boyden S. (1962). The chemotactic effect of mixtures of antibody and antigen on polymorphonuclear leucocytes. J. Exp. Med..

[36] Todaro G.J., Lazar G.K., Green H. (1965). The initiation of cell division in a contact-inhibited mammalian cell line. J. Cell Physiol..

[37] Liang C.C., Park A.Y., Guan J.L. (2007). In vitro scratch assay: A convenient and inexpensive method for analysis of cell migration in vitro. Nat. Protoc..

[38] van Meerloo J., Kaspers G.J., Cloos J. (2011). Cell sensitivity assays: The MTT assay. Methods Mol. Biol..

[39] Bratton B.A., Maly I.V., Hofmann W.A. (2019). Effect of polyunsaturated fatty acids on proliferation and survival of prostate cancer cells. PLoS ONE.

